# A Pilot Study of Bone Marrow Transplantation in a GALT‐Null Rat Model of Classic Galactosemia

**DOI:** 10.1002/jmd2.70037

**Published:** 2025-07-11

**Authors:** Shauna A. Rasmussen, Madelyn M. Seemiller, Ingrid Smith, Madeleine Wilson, Jennifer M. I. Daenzer, Keenan Wiggins, Judith L. Fridovich‐Keil

**Affiliations:** ^1^ Department of Human Genetics, Emory University School of Medicine Emory University Atlanta Georgia USA; ^2^ Emory College of Arts and Sciences Emory University Atlanta Georgia USA; ^3^ Department of Biology Emory University Atlanta Georgia USA

**Keywords:** bone marrow transplantation, galactosemia, GALT‐null, metabolic efficacy, rat model

## Abstract

Classic galactosemia (CG) is a rare inborn error of metabolism with substantial unmet medical need. Early detection, often by population newborn screening, enables immediate and life‐long dietary restriction of galactose, which is the current standard of care. This treatment minimizes or prevents the potentially lethal acute symptoms of disease in infants but fails to prevent the long‐term developmental complications experienced by most patients later in childhood. Many possible approaches to improved intervention have been proposed, ranging from small molecule inhibitors or effectors to chaperones to DNA or RNA‐based gene therapy, among others. Here, we describe the results of a pilot study testing the potential efficacy of GALT+ bone marrow transplantation (BMT) as a candidate intervention in a GALT‐null rat model of CG. Specifically, we pre‐treated adolescent GALT‐null rats with busulfan for myeloablation and then administered major histocompatibility complex (MHC)‐matched GFP+ bone marrow cells harvested from either GALT+ or GALT‐null donors. Successful engraftment of GALT+ but not GALT‐null cells resulted in > 50% wild‐type levels of GALT activity in red blood cells (RBC) and normalized RBC galactose‐1‐phosphate, a biomarker commonly followed in CG patients. However, GALT activity and galactose metabolites in both liver and brain samples remained essentially unchanged, demonstrating that successful GALT+ BMT in adolescent GALT‐null rats was not protective of other tissues.

**Summary**

- Successful engraftment of GALT+ bone marrow cells rescued RBC GALT activity and gal‐1P accumulation but not systemic enzymatic or metabolic abnormalities in a GALT‐null rat model of classic galactosemia.

## 1 Introduction

Bone marrow transplantation (BMT) is a well‐established intervention for disorders of hematopoietic origin (e.g., [1, 2]) that has also been used in both preclinical and clinical settings to treat conditions not typically associated with blood, such as lysosomal storage disorders [3, 4]. For example, a recent report [5] demonstrated evidence of both biochemical and clinical benefit to two patients with multiple sulfatase deficiency following BMT with cells from a healthy, matched donor.

The rationale for using BMT to treat solid tissue disorders is based on the understanding that while most cells derived from bone marrow remain in circulation, some, such as macrophages, migrate to the solid tissues. Donor cells may therefore provide cross‐correction to host cells mediated either systemically by factors in the blood or locally by cells of hematopoietic origin residing in the solid tissues. In the case of multiple sulfatase deficiency, eight of the 17 missing enzymes are predominantly lysosomal, suggesting the impact observed in the BMT study may have resulted from a transfer of donor cell‐derived lysosomal enzymes to host cells mediated via the mannose‐6 phosphate receptor pathway [6].

Here, we report the results of a small pilot study testing whether BMT with GALT+ donor cells might offer broad metabolic rescue in a rat model of classic galactosemia (CG). The missing enzyme in CG, galactose‐1‐phosphate uridylyltransferase (GALT), is not lysosomal [7], and therefore not easily transferred from donor to recipient cells. Nonetheless, we reasoned that having GALT+ cells circulating in the blood, and perhaps also scattered throughout the solid tissues, might provide a “sink” to deplete the galactose metabolites that would otherwise accumulate. Our results demonstrate that successful bone marrow transplant indeed restored GALT activity to a large fraction of RBCs and normalized the gal‐1P level in those cells. However, testing other tissues, including liver and brain, revealed that the correction was not systemic. This result serves as a cautionary tale that unless procedural changes dramatically improve access of bone marrow‐derived donor cells to solid tissues in the recipient, BMT may not be a promising option for treatment of CG.

## 2 Materials and Methods

### 2.1 Human Subject

The single patient described here is an adult with classic galactosemia who enrolled in Emory IRB protocol 00024933 (PI: JL Fridovich‐Keil) after appropriate informed consent. This patient received a bone marrow transplant as treatment for leukemia. Medical records were obtained with written authorization.

### 2.2 Rat Husbandry

All animal procedures, including maintenance and breeding, were approved by the Emory IACUC (PROTO201700095; PI: JL Fridovich‐Keil) and conducted with oversight of the Emory Division of Animal Resources (DAR). Rat pups were weaned at P24 into sterile cages provided by the DAR. All post‐wean rats had *ad libitum* access to drinking water and solid chow (LabDiet 5053) that included approximately 1% of calories from galactose.

### 2.3 Genotyping Animals at the *GFP* , *Galt*, and *RT1* (MHC) Loci

See Supplemental Methods.

### 2.4 Busulfan Pre‐Treatment

See Supplemental Methods.

### 2.5 Isolation and Administration of Bone Marrow Cells

See Supplemental Methods.

### 2.6 Monitoring Engraftment of GFP+ Bone Marrow Cells in Rats by Flow Cytometry

#### 2.6.1 Blood sample collection

Small blood samples were collected from the tail vein at four‐, six‐, and eight‐weeks post‐transplant, with a final blood sample collected at 10‐weeks post‐transplant during euthanasia and tissue harvest. For each tail vein blood draw, rats were anesthetized by isoflurane inhalation, and the tail vein was dilated by contact with 40°C–45°C water for 30 s. Aseptic techniques were then used to collect 500 μLs of whole blood from the tail vein into a sterile syringe; the blood was then immediately transferred into a heparin lined 1.5 mL Eppendorf tube, which was inverted eight–10 times for mixing. Blood cells were pelleted at 4°C by centrifugation for 15 min at 2000 x g (4600 rpm) in an Eppendorf 5415D microfuge. The plasma was removed, aliquoted, and stored at −80°C until extraction for metabolite quantitation. The cell pellet was resuspended in 1 mL of 1X PBS and centrifuged at 4°C at 2000 x g (4600 rpm) for 15 mins. The wash supernatant was discarded, and the cell pellet was aliquoted and either processed immediately for flow cytometry or stored at −80°C for later biochemical studies. Details explaining how blood and bone marrow samples were prepared for flow cytometry are presented in Supplemental Methods.

### 2.7 Quantifying GALT Activity and Galactose, Galactitol, and Gal‐1P Metabolites in Samples of Blood, Liver, and Brain From Treated and Control Rats

#### 2.7.1 GALT activity

GALT activity was quantified in samples of RBC hemolysate, liver, and brain as described previously [8] starting with 25–30 mg of solid tissue or 100 μLs of hemolysate. Individual reactions using samples from wild‐type rats included 40 μg Hb (if RBC), 2 μg protein (if liver), or 10 μg protein (if brain). Individual reactions using samples from GALT‐null rats included 100 μg Hb (if RBC), 10 μg protein (if liver), or 40 μg protein (if brain). If a reaction revealed activity outside the linear range, defined as < 80% starting gal‐1P substrate remaining at the end of the reaction, the assay was repeated using appropriately diluted sample.

#### 2.7.2 Metabolites

Galactose, galactitol, and gal‐1P were quantified in samples of hemolysate, liver, and brain as described previously [8]. Galactose and galactitol were also quantified in samples of plasma as described previously [8].

## 3 Results

### 3.1 Breeding Rats to Serve as Bone Marrow Donors and Recipients

To facilitate easy tracking of donor cells by flow cytometry, we crossed a male SD‐Tg(UBC‐EGFP)2BalRrrc [9] Sprague–Dawley rat carrying a ubiquitously expressed green fluorescent protein (*GFP*) transgene into our colony. We bred rats heterozygous for this *GFP* transgene [9] and also our GALT‐null allele [10], but homozygous for the same *RT1* allele at the *MHC* locus [11]. Crossing these male and female breeder rats produced the donor and recipient rats needed for this study (Supplemental Figure S1). Specifically, donor rats were GFP+ (either homozygous or heterozygous) and either GALT+ (test) or GALT‐null (control). Recipient pups were null for both GFP and GALT. Genotypes at all relevant loci were confirmed as described in Supplemental Methods.

### 3.2 Successful Engraftment of Donor Bone Marrow Cells in GALT‐Null Recipient Rats

To facilitate BMT engraftment, GALT‐null rats were conditioned starting at P30 with busulfan for myeloablation (see Supplemental Methods) and then administered GFP+ bone marrow cells harvested from either GALT+ or GALT‐null donor rats, as described in Supplemental Methods. Of the seven recipient rats transplanted for this pilot study, two died at 15‐ and 26 days following transplant, respectively. Both of these rats had received busulfan pretreatment and donor cell transplant in parallel with the other treated rats, and all recipient and donor rats were MHC‐identical. We suspect these two rats may have died as a result of the myeloablation, but cannot rule out other possible causes. The remaining five treated rats recovered, grew, and demonstrated sustained improvement.

**FIGURE 1:**
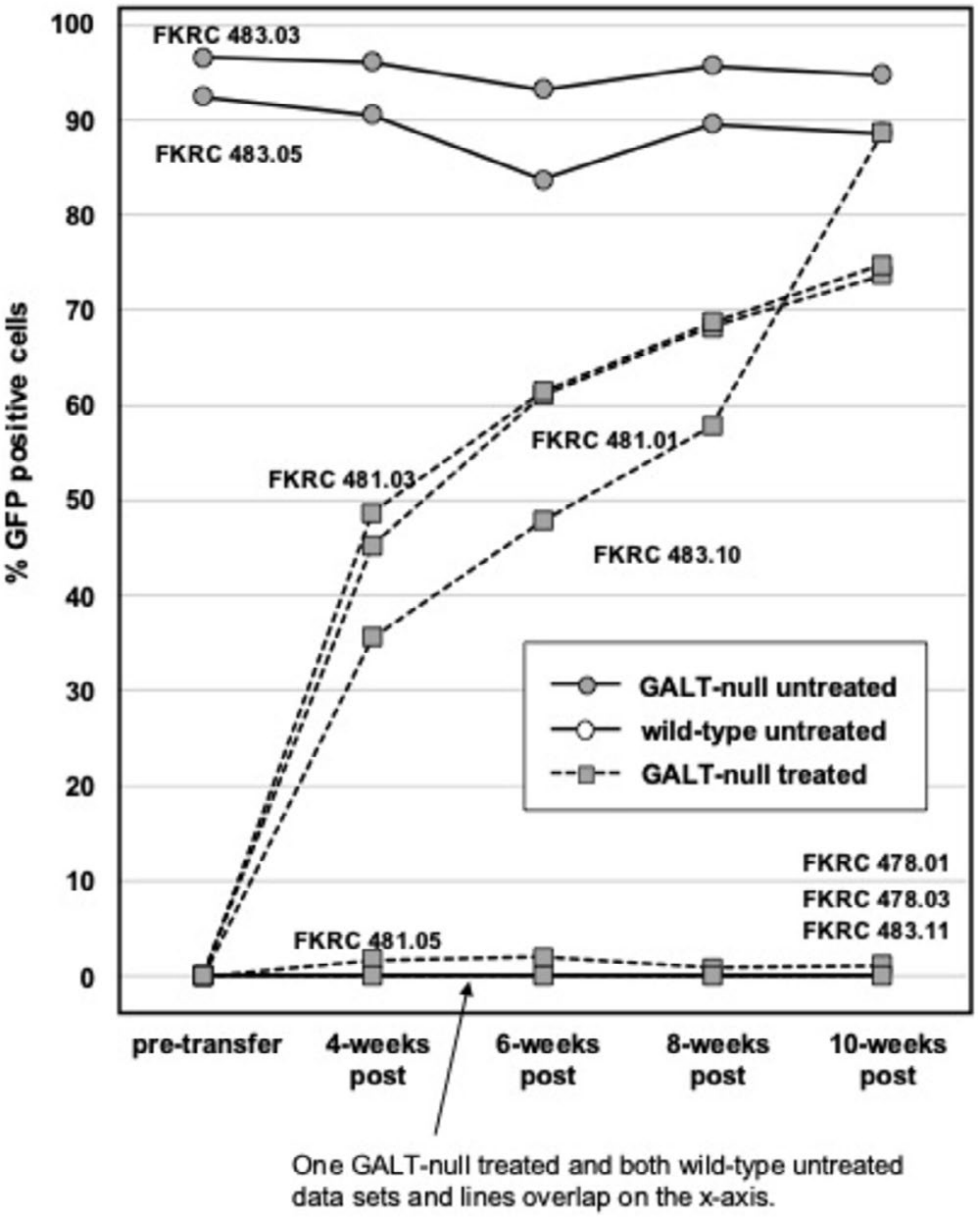
Engraftment of GFP+ cells following bone marrow transplantation in busulfan‐treated GFP‐null, GALT‐null recipient rats. FKRC numbers indicate rat ID codes; digits before the decimal point indicate the litter and digits following the decimal point indicate the individual pup number. Circles connected by solid lines represent untreated control rats, of which two were GFP+ and two were GFP‐null. Squares connected by dashed lines represent GFP‐null rats transplanted with GFP+ donor bone marrow. The symbols and connecting lines representing the two GFP‐null wild‐type rats are hidden along the X‐axis behind symbols and solid connecting lines representing one of the transplanted rats that demonstrated poor engraftment.

To track the progress of donor cell engraftment in the five surviving rats, we collected tail vein blood samples just before the first busulfan treatment and then again at four‐, six‐, and eight‐weeks post‐transplant. As controls, we also included two samples from untreated GALT+, GFP‐null rats and two samples from GALT‐null, GFP+ rats. At 10 weeks post‐transplant, the five recipient and four control rats were all euthanized for harvest of blood, bone marrow, liver, and brain. The percentage of GFP+ cells detected by flow cytometry in all blood samples is presented in Figure 1. Visual flow cytometry results of blood and bone marrow samples from two recipient rats—one receiving GALT+ donor cells (FKRC481.01) and the other receiving GALT‐null donor cells (FKRC483.10) —are presented in Supplemental Figure S2.

Of the five transplanted rats harvested at 10 weeks post‐transfer, four (FKRC ID#s 481.01, 481.03, 481.05, and 483.11) received GALT+ donor cells and one (FKRC483.10) received GALT‐null donor cells. Three of the five rats (two receiving GALT+ cells and one receiving GALT‐null cells) showed strong engraftment, with > 70% of white blood cells demonstrating GFP signal at 10 weeks post‐transplant. The remaining two rats, both recipients of GALT+ donor cells, showed only poor engraftment, with 1.12% and 0.01% of white blood cells demonstrating GFP signal at 10 weeks post‐transplant, respectively (Figure 1).

Of note, despite the poor engraftment seen in these two rats, both the volumes of packed red blood cells per milliliter of whole blood and the hemoglobin levels seen per volume of packed red blood cells at 10 weeks post‐transplant did not differ substantially among the five transplanted rats or the four untreated controls (data not shown). These results suggest that bone marrow in the two rats showing poor engraftment had repopulated their RBCs to normal levels following the busulfan treatment, albeit ostensibly with cells derived from endogenous bone marrow that had escaped myeloablation rather than from donor cells.

Finally, we measured GALT activity in RBCs harvested from each of the nine rats euthanized at the 10‐week post‐transplant time point (Table S1). As expected, both untreated wild‐type (GALT+) control rats (FKRC ID#s 478.01 and 478.03) showed strong GALT activity in RBCs (2.26 and 2.18 pmol UDP‐gal/μg Hb/min, respectively), whereas both untreated GALT‐null control rats (ID#s 483.03 and 483.05) showed no detectable GALT activity in RBCs (−0.14 and 0.02 pmol UDP‐gal/μg Hb/min, respectively). The single GALT‐null rat successfully transplanted with GALT‐null bone marrow cells (FKRC ID# 483.10) also showed no detectable GALT activity in RBCs (0.01 pmol UDP‐gal/μg Hb/min). In contrast, both GALT‐null rats successfully transplanted with GALT+ bone marrow cells (FKRC ID#s 481.01 and 481.03) demonstrated more than half the wild‐type level of GALT activity in RBCs (1.21 and 1.37 pmol UDP‐gal/μg Hb/min, respectively). The two GALT‐null rats that showed poor engraftment with GALT+ donor cells by GFP signal (FKRC ID#s 481.05 and 483.11) also showed intermediate GALT activity in hemolysates (Table S1).

### 3.3 Impact of Successful GALT+ Bone Marrow Transplant on RBC Gal‐1P Levels

Next, we tested the metabolic efficacy of successful GALT+ bone marrow cell engraftment in GALT‐null rats by measuring RBC gal‐1P in blood. Four patterns were clear (Table 1). First, as expected, untreated GALT‐null rats showed orders of magnitude higher RBC gal‐1P than did wild‐type rats. Second, RBC gal‐1P levels diminished gradually over the time course of the experiment, even in untreated GALT‐null rats. This was expected, as is explained in the Discussion. Third, and most promising, GALT‐null rats successfully transplanted with GALT+ bone marrow showed RBC gal‐1P levels comparable to the levels seen in wild‐type controls. Finally, as expected, GALT‐null rats that showed only poor engraftment with GALT+ bone marrow cells also showed only partial correction of RBC gal‐1P, and the single surviving rat successfully engrafted with GALT‐null donor bone marrow cells showed RBC gal‐1P comparable to the levels seen in untreated GALT‐null rats (Table 1).

**TABLE 1:** Longitudinal measures of GFP+ cell engraftment and RBC gal‐1P in samples from 5 BMT transplant recipients and 4 controls in this pilot study.

Rat FKRC ID#	Recipient *GALT* and GFP genotypes	Treatment group	RBC Gal‐1P prior to treatment (% GFP+ cells)	RBC Gal‐1P at 4‐weeks post (% GFP+ cells)	RBC Gal‐1P at 6‐weeks post (% GFP+ cells)	RBC Gal‐1P at 8‐weeks post (% GFP+ cells)	RBC Gal‐1P at 10‐weeks post (% GFP+ cells)
478.01	Wild‐type (no GFP)	no BMT	4.48 (0)	1.80 (0)	2.48 (4.12x10E‐3)	1.27 (0)	0.39 (0.01)
478.03	Wild‐type (no GFP)	no BMT	3.71 (0)	14.61 (0.04)	2.34 (0.01)	5.06 (2.66x10E‐3)	1.19 (5.34x10E‐3)
483.03	GALT‐null (GFP+)	no BMT	1128.04 (96.5)	576.65 (96)	332.39 (93.2)	346.72 (95.7)	328.09 (94.7)
483.05	GALT‐null (GFP+)	no BMT	733.83 (92.4)	937.41 (90.5)	148.58 (83.7)	359.80 (89.5)	273.78 (88.6%)
481.01	GALT‐null (no GFP)	BMT with GALT+ donor cells	894.24 (0.08)	163.04 (45.2)	9.82 (61)	12.18 (68.2)	2.63 (73.7)
481.03	GALT‐null (no GFP)	BMT with GALT+ donor cells	no data (1.19x10E‐3)	108.54 (48.7)	15.72 (61.4)	4.68 (68.7)	0.96 (74.7)
481.05	GALT‐null (no GFP)	BMT with GALT+ donor cells	1188.42 (0)	992.93 (1.71)	390.04 (2.01)	245.96 (0.88)	110.83 (1.12)
483.11	GALT‐null (no GFP)	BMT with GALT+ donor cells	897.10 (3.11x10E‐3)	63.61 (0.01)	105.40 (0)	147.53 (3.55x10E‐3)	136.87 (0.01)
483.10	GALT‐null (no GFP)	BMT with GALT‐null donor cells	696.97 (0)	347.79 (35.6)	271.14 (47.8)	365.35 (57.8)	244.40 (88.6)

### 3.4 Successful Engraftment of GALT+ Bone Marrow Cells Showed Little to no Impact on Liver or Brain GALT Activity or Metabolites

Finally, we asked whether the successful transplant of GALT‐null rats with GALT+ bone marrow cells would show any detectable impact on GALT activity in the liver or brain, and any metabolic efficacy on galactose metabolites in plasma, liver, or brain. The short answer was no. GALT activity levels in both the liver and brain remained near baseline even in the two rats showing > 70% GFP+ (donor) cells in blood (FKRC 481.01 and FKRC 481.03; Table S2). Among metabolites, galactose showed trace if any improvement in RBC or liver, and no improvement in plasma or brain (Table S3). Galactitol showed no improvement in any sample type (Table S4). Finally, despite showing a dramatic improvement in RBC following successful engraftment of GALT+ bone marrow cells, gal‐1P showed no detectable improvement in liver or brain (Table 2).

**TABLE 2:** Gal‐1P levels in RBC, liver, and brain samples from transplanted and control rats harvested at the 10‐week post‐transplant time point.

Rat FKRC ID#	Recipient *GALT* and GFP genotypes	Treatment group (% GFP+ cells in blood at 10‐weeks post‐transplant)	Gal‐1P in RBC pmol/μL	Gal‐1P in liver pmol/μg	Gal‐1P in brain pmol/μg
478.01	Wild‐type (no GFP)	no BMT (0.01%)	0.69	41.83	0.27
478.03	Wild‐type (no GFP)	no BMT (5.3x10E‐3%)	0.54	59.81	1.36
483.03	GALT‐null (GFP+)	no BMT (94.7%)	312.53	865.46	93.84
483.05	GALT‐null (GFP+)	no BMT (88.6%)	277.48	1064.74	114.77
481.01	GALT‐null (no GFP)	BMT with GALT+ donor cells (73.7%)	2.63	804.62	73.70
481.03	GALT‐null (no GFP)	BMT with GALT+ donor cells (74.7%)	0.96	928.46	84.01
481.05	GALT‐null (no GFP)	BMT with GALT+ donor cells (1.12%)	110.83	1490.79	72.30
483.11	GALT‐null (no GFP)	BMT with GALT+ donor cells (0.01%)	136.87	1250.91	91.61
483.10	GALT‐null (no GFP)	BMT with GALT‐null donor cells (88.6%)	244.40	1026.47	80.78

## 4 Discussion

This limited pre‐clinical study serves as a cautionary tale that bone marrow transplant, as modeled here, may not be an effective approach to restore galactose metabolism in a GALT‐null recipient. Specifically, in our GALT‐null rats that were successfully engrafted with GALT+ donor bone marrow cells, we observed a striking rise in RBC GALT activity and a corresponding drop in RBC gal‐1P to essentially normal levels. However, we did not see comparable correction of either galactose or galactitol in RBC or plasma, or correction of galactose, galactitol, or gal‐1P in liver or brain. In short, the presence of GALT+ cells in bone marrow and blood did not appear to provide metabolic cross‐correction to other tissues. To our knowledge, this is the first time GALT+ bone marrow transplant has been tested as a potential intervention for GALT deficiency in an animal model of CG.

We are also aware of a single patient with CG who developed leukemia as an adult and received a therapeutic BMT. To be clear, the BMT in this patient was intended as a treatment for leukemia, not galactosemia. Because the donor was GALT+, successful engraftment not only treated the leukemia but also conferred normal GALT activity in RBCs (25.3 nmol/h/mg Hb, reference range > =24.5), prompting the question—Does this patient still have galactosemia? Unfortunately, neither pre‐ nor post‐transplant metabolite values were available, and health complications from the patient's leukemia prevented further testing. Extending from our results in rats, we predict that post‐transplant, this patient would have shown normalized levels of gal‐1P in RBC but continued elevation of galactitol in urine.

In the rat model, that successful engraftment of GALT+ bone marrow cells corrected RBC gal‐1P but not RBC galactose or galactitol seems counterintuitive but may reflect the reality that gal‐1P is a strictly intracellular metabolite, whereas both galactose and galactitol cross the cell membrane. The galactose and galactitol that accumulated in the many GALT‐null tissues of our transplanted rats therefore contributed to increased galactose and galactitol in the plasma and RBCs of those animals. In contrast, any gal‐1P that accumulated in the many GALT‐null tissues was locked in those tissues and unable to contribute to the gal‐1P in RBCs. Of course, other explanations may also apply.

Another result presented here that requires explanation is the approximately two to three‐fold drop in RBC gal‐1P detected in untreated GALT‐null rats between the first and last time points in this experiment (Table 1). This drop in gal‐1P with increasing age is fully consistent with prior reports from both galactosemia patients [12] and GALT‐null rats [8, 10] and likely reflects a combination of factors. First, dietary galactose exposure in the rats described here dropped from about 3% of calories (pre‐wean) to about 1% of calories at weaning, which occurred approximately one week before the first blood sample for this study was collected. Second, endogenous galactose production is known to decrease with age in CG patients [13, 14, 15] and therefore may also decrease with age in rats. Finally, prior studies in GALT‐null rats have documented increased capacity with age of a GALT‐independent, UDP‐glucose/galactose pyrophosphorylase (UGP)‐mediated “bypass” pathway for galactose metabolism [8].

### 4.1 Limitations

While carefully designed and conducted, this pilot study in rats nonetheless had numerous limitations. Perhaps the most obvious limitation was size. Due to financial and other constraints, we only transplanted seven rats, and of those only five survived. Each comparison group therefore included only one or two rats, precluding statistical analysis and preventing the interpretation of potentially subtle differences. Similarly, we had only limited information about a single patient with CG who received a GALT+ BMT.

Furthermore, in the rat model, we transplanted recipients at a single age, 34–35 days, leaving open the possibility that results may have been different had we conducted the procedure using older or younger recipients or donors [16]. This point may be especially relevant given the potentially limited early window of developmental time during which bone marrow‐derived cells are able to contribute to resident cell populations in the liver, brain, and perhaps other solid tissues [17]. Finally, it is possible that a different pre‐treatment regimen with busulfan, radiation, or other agents might have improved access of donor bone marrow‐derived cells to compartments beyond the circulating blood [18].

## Conflicts of Interest

The authors declare no conflicts of interest.

## Supporting information


**Figure S1.** Diagram illustrating transplantation strategy. Rats were bred to homozygosity at the *RT1* (major histocompatibility) locus to minimize the risk of graft/host rejection. All donors were GFP+ and either GALT+ or GALT‐null; all recipients were both GFP‐null and GALT‐null.

**Figure S2.** Flow cytometry results of blood and bone marrow samples from two representative GALT‐null rats (FKRC481.01 and FKRC483.10) transplanted for this study. The small square within each field indicates cells defined as GFP+.

Data S1.

**Table S1.** Donor cell engraftment and RBC GALT activity at 10‐weeks post‐BMT in five transplanted rats and four untreated controls.

**Table S2.** GALT activity in liver and brain samples from transplanted and control rats harvested at the 10‐week post‐transplant time point.

**Table S3.** Galactose levels in RBC, plasma, liver, and brain samples from transplanted and control rats harvested at the 10‐week post‐transplant time point.

**Table S4.** Galactitol levels in RBC, plasma, liver, and brain samples from transplanted and control rats harvested at the 10‐week post‐transplant time point.

## Data Availability

The data that supports the findings of this study are available in the Tables, Figures, and Supporting Information of this article.
