# Supplementary figures and images for: A Pilot Study of Bone Marrow Transplantation in a GALT‐Null Rat Model of Classic Galactosemia
Source: JIMD Rep. 2025 Jul 11;66(4):e70037. doi: 10.1002/jmd2.70037 (PMC12254465; doi:10.1002/jmd2.70037)

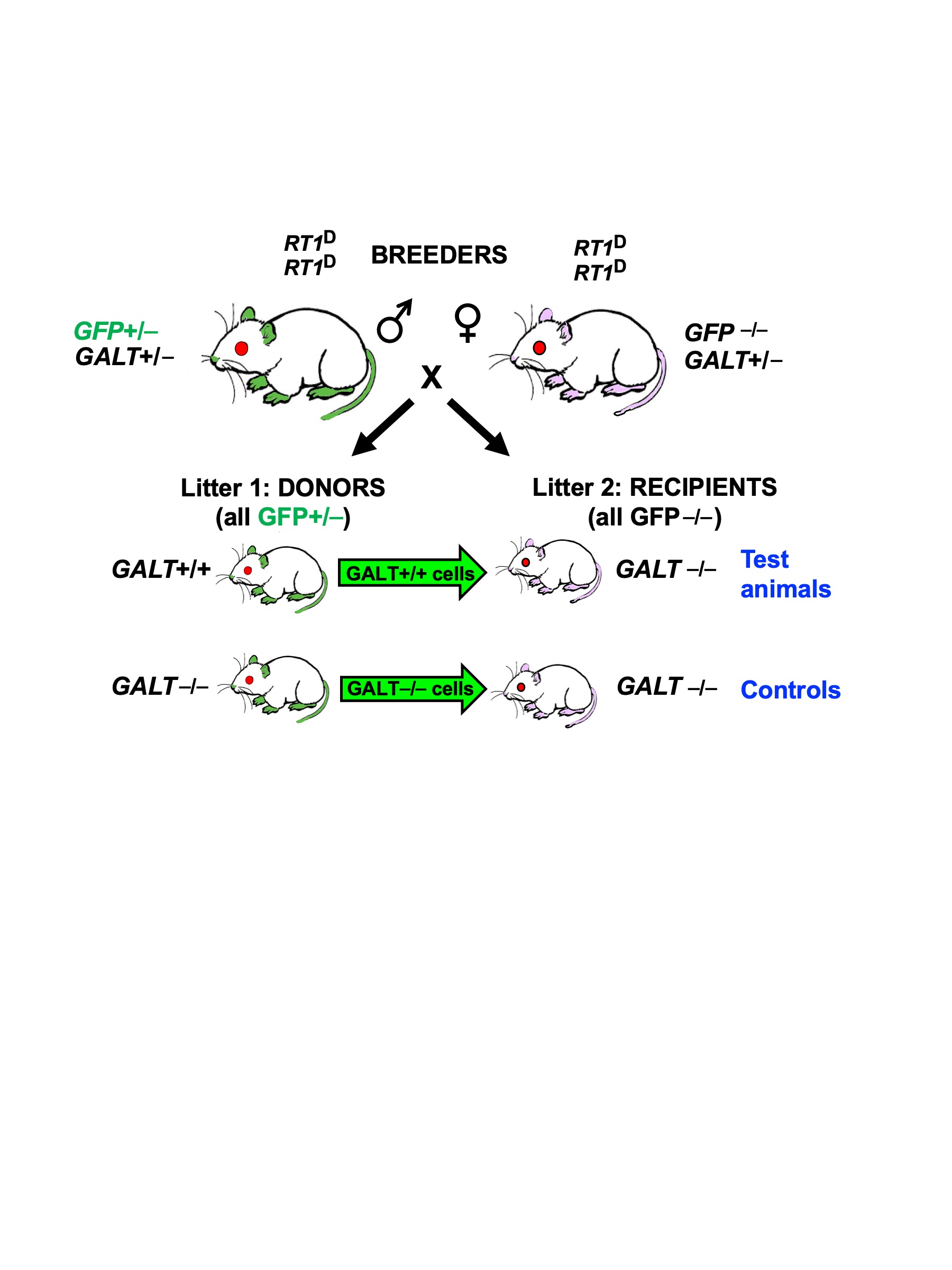

Supplement: Supplementary file 1 — Figure S1. Diagram illustrating transplantation strategy. Rats were bred to homozygosity at the RT1 (major histocompatibility) locus to minimize the risk of graft/host rejection. All donors were GFP+ and either GALT+ or GALT‐null; all recipients were both GFP‐null and GALT‐null. [file JMD2-66-e70037-s002.jpg]

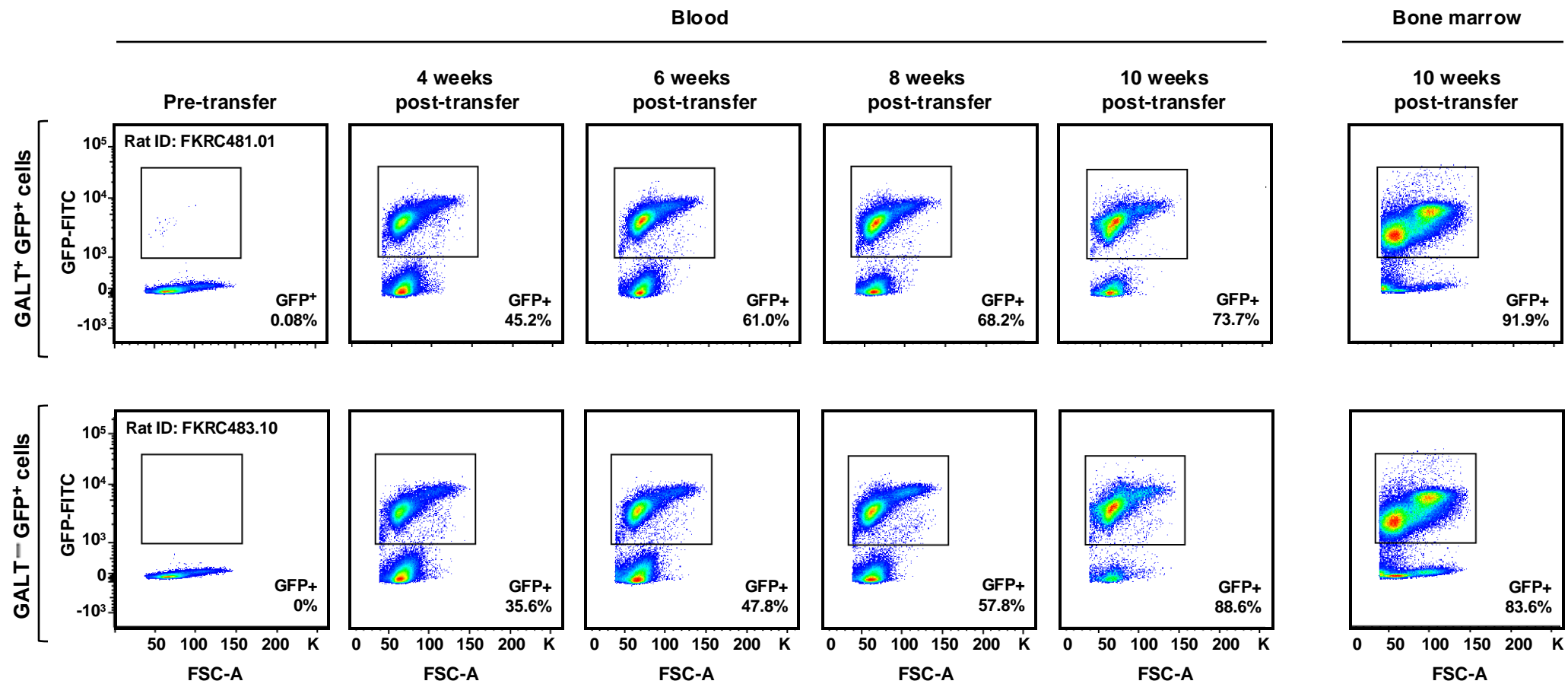

Supplement: Supplementary file 2 — Figure S2. Flow cytometry results of blood and bone marrow samples from two representative GALT‐null rats (FKRC481.01 and FKRC483.10) transplanted for this study. The small square within each field indicates cells defined as GFP+. [file JMD2-66-e70037-s005.pdf]
