# Supplementary material for: A Pilot Study of Bone Marrow Transplantation in a GALT‐Null Rat Model of Classic Galactosemia
Source: JIMD Rep. 2025 Jul 11;66(4):e70037. doi: 10.1002/jmd2.70037 (PMC12254465; doi:10.1002/jmd2.70037)
Supplement: Supplementary file 3 — Data S1. [file JMD2-66-e70037-s004.pdf]

Supplemental Methods for Rasmussen, Seemiller, et al.

Genotyping animals at the *GFP*, *Galt*, and *RT1* (MHC) loci

GFP: The green fluorescent protein (*GFP*) transgene used here was introduced into our GALT-null rats by breeding a male SD-Tg(UBC-EGFP)2BalRrrc [1], purchased from the Rat Resource and Research Center (RRRC# 0065), with a GALT-null female from our colony. Pups were screened for presence of the *GFP* transgene by PCR-based genotyping using the following primer sequences: R52 int 1F Sequence: 5'-AGC AAT GAA TAG CCT CTC TCC T-3'; R52 int 1R Sequence: 5'-CCC ATA TGT GCC AAG CAC TTT ACC-3'; U3r-0 Sequence: 5'-GTC TGA AGG GAT GGT TGT AGC TGT-3', as described previously [1]. Expression of GFP was further confirmed by visual inspection of the rats using an ultraviolet blacklight with filter glasses.

Galt: Rats were genotyped for presence or absence of the GALT-null ($Galt^{M3}$) allele by Transnetyx, Inc. (https://www.transnetyx.com/), as described previously [2].

RT1: Rats were genotyped at the *RT1* (major histocompatibility, *MHC*) locus [3] to enable selective breeding of rats to *RT1* homozygosity while maintaining the needed genotypes at the *GFP* and *Galt* loci. Specifically, known polymorphic regions of the *RT1* locus were PCR amplified using the following primer sets and sequenced to reveal the alleles present in each rat: primer set 1 (AF3 5'-CTACTACAACCAGAGCGAG-3'; AR3 5'-GCATCACCCAGCTGAG-3'), primer set 2 (M2F2 5'- CACCAAGGACCCATGTG-3'; M2R2 5'-CTGCCTCATTGTTGAAATGC-3'), and primer set 3 (CE16F2 5'-CATGGAAGTCGGCTACG-3'; CE16R2 5'-CTTGTTCCGGGTGATCC-3'). Crosses were then arranged between rats carrying the same *RT1* alleles until homozygosity at the *RT1* locus was achieved.

Preparing blood samples for flow cytometry

Fresh heparinized whole blood was prepared for flow cytometry using eBioscience 1X RBC Lysis Buffer as recommended by the manufacturer (Invitrogen/ Thermo Fisher Scientific, https://www.thermofisher.com/order/catalog/product/00-4300-54). We mixed 100 µLs heparinized whole blood with 2 mLs of 1X RBC Lysis Buffer (Multi-species), agitated briefly by pulse vortex to mix, and then incubated at room temperature for 4-10 minutes. We then centrifuged each sample at 500 x g for 5 minutes at room temperature, discarded the supernatant, resuspended the pellet in 2 mLs of Flow Cytometry Staining Buffer (2% FBS in 1X PBS) and centrifuged again at 500 x g for 5 minutes at room temperature. Finally, we discarded the supernatant, resuspended the cell pellet in 200 µLs of Flow Cytometry Staining Buffer, and analyzed the sample using an LSR Fortessa X-20 (BD Biosciences). All flow cytometry data were analyzed with FlowJo v10. The following gating strategy preceded all flow cytometry analyses presented. Cells were gated on (1) lymphocytes (forward light scatter [FSC]–area X side scatter [SSC]–area), (2) singlets (FSC-width X FSC-height and SSC-width X SSC-height), and (3) GFP positive cells (FITC X FSC).

Preparing bone marrow samples for flow cytometry:

At 10-weeks post-transplant, all experimental rats were euthanized and bone marrow was collected as described above. Following collection, bone marrow cells were pelleted by centrifugation at 500 x g for 5 minutes at room temperature and then resuspended in 5 mLs of 1X RBC Lysis Buffer (Multi-species, as above). After a 4-5 minute incubation period at room temperature, the cells were again pelleted by centrifugation at 500 x g for 5 minutes at room temperature and then washed and resuspended in Flow Cytometry Staining Buffer as described above for blood cells.

Busulfan pre-treatment

Busulfan was prepared fresh each day for administration as follows: under sterile conditions, 30 mg of busulfan (Millipore Sigma B2635) was transferred into a sterile 1.5 mL tube and dissolved in 1 mL of 100% DMSO (Millipore Sigma D2650). This solution was then diluted with 1X PBS (Corning #21-040-CV) at 37°C to a final volume of 10 mLs and sterilized by passing through a 0.2 μm syringe filter.

30-day-old rats intended as recipients of donor bone marrow were administered 25 mg/kg busulfan by intraperitoneal (IP) injection on each of 2 successive days using a sterile 1-mL 25G tuberculin syringe. Following busulfan administration, rats were maintained in sterile cages with *ad libitum* access to chow and sterile water containing 1.1 mg/mL Neomycin (Millipore Sigma N5285) and 1000 U/mL Polymyxin B Sulfate (Millipore Sigma P0972) to protect against infection.

Isolation and administration of bone marrow cells

Isolation of bone marrow cells: Bone marrow cells were isolated from young adult donor rats (at P30) essentially as described previously for mice [4, 5]. First, the donor was anesthetized with isoflurane and euthanized via exsanguination. Under sterile conditions, we then removed the femurs and placed them in a tissue culture dish. Using sterile forceps and small scissors, we removed muscle and fibrous tissue from each bone and then wiped the bones clean of any remaining loose tissue using a Kimwipe saturated with 70% ethanol. Next, we pipetted 10-15 mLs RPMI complete medium into a sterile cell culture dish. Using a scalpel or sharp scissors, we removed both ends of each femur and then drew up 10 mLs RPMI complete medium into a 10 mL syringe fitted with a 23-gauge needle. Using sterile forceps to hold each bone above the sterile cell culture dish containing medium, we carefully flushed marrow from bones into the cell

culture dish using the syringe. We repeated the flushing step until each bone appeared white and translucent, indicating that most of the marrow had been removed.

Next, we placed a sterile 70 µm cell strainer on top of a sterile 50 mL conical tube, held upright in a tube rack. Using a sterile serological pipet, we transferred the bone marrow slurry from the cell culture dish into the strainer. As needed, we added more RPMI complete medium to the cell culture dish to transfer any remaining marrow into the strainer. Next, we discarded the strainer and pelleted the filtered cell suspension by centrifugation at 600 x g at 4°C for 4 minutes and discarded the supernatant. The cell pellet was then resuspended in 50 mLs RPMI medium and passed through a second sterile 70 µm cell strainer into a new 50 mL conical tube, and once again cells were pelleted by centrifugation at 600 x g at 4°C for 4 minutes. Next, we resuspended the cell pellet in 10 mLs sterile 1X PBS and removed 100 µL of the suspension, mixing it with 1 mL 1X RBC lysis buffer (eBioscience™ 10X RBC Lysis Buffer, Multi-species) in a sterile 15cc tube to lyse the red cells. We incubated this suspension at room temperature for 4-5 minutes with occasional gentle rocking, then added 2 mLs sterile 1X PBS and centrifuged at 600 x g for 4 minutes at 4°C, discarding the supernatant. Finally, we resuspended the cell pellet in 10mLs sterile 1X PBS, pipetting up and down as needed to disrupt the pellet, and counted the cells with a hemocytometer or automated cell counter.

Injection of donor bone marrow cells: Approximately 72 hours after the second busulfan injection, recipient rats were administered $5x10^7$ donor bone marrow cells suspended in 200 µL sterile 1X PBS. First, rats were anesthetized by isoflurane inhalation and aseptic techniques were used to prepare the tail vein for injection. Next, the cell suspension was drawn up into a 1-mL syringe fitted with a 27G needle, air was removed by inverting the syringe and pressing gently on the plunger, and then the needle was inserted into the tail vein, with placement confirmed by a blood flash into the needle. Finally, donor cells were injected into the vein. The needle was held in place for approximately 10 seconds after the injection to prevent leakage

and then removed with gentle pressure applied to the injection site with a gloved finger on a gauze pad until any bleeding had stopped. Post-injection, rats were housed in sterile cages for one week, after which time they were returned to standard housing conditions, although they were maintained on antibiotic water (see above) for a full month post-transplant.

References (for Supplemental Methods)

1. Men, H., B.A. Bauer, and E.C. Bryda, *Germline transmission of a novel rat embryonic stem cell line derived from transgenic rats.* Stem Cells Dev, 2012. **21**(14): p. 2606-12.
2. Rasmussen, S.A., et al., *A galactose-1-phosphate uridylyltransferase-null rat model of classic galactosemia mimics relevant patient outcomes and reveals tissue-specific and longitudinal differences in galactose metabolism.* J Inherit Metab Dis, 2020. **43**(3): p. 518-528.
3. Walter, L., *Nomenclature report on the major histocompatibility complex genes and alleles of the laboratory rat (Rattus norvegicus).* Immunogenetics, 2020. **72**(1-2): p. 5-8.
4. Madaan, A., et al., *A stepwise procedure for isolation of murine bone marrow and generation of dendritic cells.* Journal of Biological Methods, 2014. **1**(1).
5. Liu, X. and N. Quan, *Immune Cell Isolation from Mouse Femur Bone Marrow.* Bio Protoc, 2015. **5**(20).
