# Supplementary material for: A Pilot Study of Bone Marrow Transplantation in a GALT‐Null Rat Model of Classic Galactosemia
Source: JIMD Rep. 2025 Jul 11;66(4):e70037. doi: 10.1002/jmd2.70037 (PMC12254465; doi:10.1002/jmd2.70037)
Supplement: Supplementary file 4 — Table S1. Donor cell engraftment and RBC GALT activity at 10‐weeks post‐BMT in five transplanted rats and four untreated controls. [file JMD2-66-e70037-s001.pdf]

Supplemental Table 1: Donor cell engraftment and RBC GALT activity at 10-weeks post-BMT in 5 transplanted rats and 4 untreated controls.

Rat FKRC ID#	Recipient genotype for GALT and GFP	Treatment group	% GFP+ white cells at 10-weeks post-transplant	RBC GALT activity at 10-weeks post-transplant (pmol UDP-gal/µg Hb/min)
478.01	Wild-type (no GFP)	no BMT	0.01	2.26
478.03	Wild-type (no GFP)	no BMT	5.3x10E-3	2.18
483.03	GALT-null (GFP+)	no BMT	94.7	-0.14
483.05	GALT-null (GFP+)	no BMT	88.6	0.02
481.01	GALT-null (no GFP)	BMT with GALT+, GFP+ donor cells	73.7	1.21
481.03	GALT-null (no GFP)	BMT with GALT+, GFP+ donor cells	74.7	1.37
481.05	GALT-null (no GFP)	BMT with GALT+, GFP+ donor cells	1.12	0.75
483.11	GALT-null (no GFP)	BMT with GALT+, GFP+ donor cells	0.01	0.66
483.10	GALT-null (no GFP)	BMT with GALT-null, GFP+ donor cells	88.6	0.01
