# Supplementary material for: A Pilot Study of Bone Marrow Transplantation in a GALT‐Null Rat Model of Classic Galactosemia
Source: JIMD Rep. 2025 Jul 11;66(4):e70037. doi: 10.1002/jmd2.70037 (PMC12254465; doi:10.1002/jmd2.70037)
Supplement: Supplementary file 5 — Table S2. GALT activity in liver and brain samples from transplanted and control rats harvested at the 10‐week post‐transplant time point. [file JMD2-66-e70037-s003.pdf]

Supplemental Table 2: GALT activity in liver and brain samples from transplanted and control rats harvested at the 10-week post-transplant time point.

Rat FKRC ID#	Recipient *GALT* and GFP genotypes	Treatment group (% GFP+ cells in blood at 10-weeks post-transplant)	GALT activity in LIVER pmol/µg protein/min	GALT activity in BRAIN pmol/µg protein/min
478.01	Wild-type (no GFP)	no BMT (0.01%)	16.72	4.17
478.03	Wild-type (no GFP)	no BMT (5.3x10E-3%)	22.5	2.1
483.03	GALT-null (GFP+)	no BMT (94.7%)	0.1	-0.06
483.05	GALT-null (GFP+)	no BMT (88.6%)	-0.3	0.02
481.01	GALT-null (no GFP)	BMT with GALT+ donor cells (73.7%)	0.57	0.02
481.03	GALT-null (no GFP)	BMT with GALT+ donor cells (74.7%)	0.5	0.05
481.05	GALT-null (no GFP)	BMT with GALT+ donor cells (1.12%)	-0.01	-0.02
483.11	GALT-null (no GFP)	BMT with GALT+ donor cells (0.01%)	-0.01	-0.02
483.10	GALT-null (no GFP)	BMT with GALT-null donor cells (88.6%)	0.3	-0.14
