# Supplementary material for: A Pilot Study of Bone Marrow Transplantation in a GALT‐Null Rat Model of Classic Galactosemia
Source: JIMD Rep. 2025 Jul 11;66(4):e70037. doi: 10.1002/jmd2.70037 (PMC12254465; doi:10.1002/jmd2.70037)
Supplement: Supplementary file 6 — Table S3. Galactose levels in RBC, plasma, liver, and brain samples from transplanted and control rats harvested at the 10‐week post‐transplant time point. [file JMD2-66-e70037-s007.pdf]

Supplemental Table 3: Galactose levels in RBC, plasma, liver, and brain samples from transplanted and control rats harvested at the 10-week post-transplant time point.

Rat FKRC ID#	Recipient *GALT* and GFP genotypes	Treatment group (% GFP+ cells in blood at 10-weeks post-transplant)	Galactose in RBC pmol/µL	Galactose in plasma pmol/µL	Galactose in liver pmol/µg	Galactose in brain pmol/µg
478.01	Wild-type (no GFP)	no BMT (0.01%)	0.23	1.60	0.07	0.74
478.03	Wild-type (no GFP)	no BMT (5.3x10E-3%)	0.17	2.98	0.15	1.57
483.03	GALT-null (GFP+)	no BMT (94.7%)	5.45	79.09	29.93	36.35
483.05	GALT-null (GFP+)	no BMT (88.6%)	9.08	24.28	112.21	27.51
481.01	GALT-null (no GFP)	BMT with GALT+ donor cells (73.7%)	2.77	23.24	57.88	15.17
481.03	GALT-null (no GFP)	BMT with GALT+ donor cells (74.7%)	1.45	27.59	81.76	39.25
481.05	GALT-null (no GFP)	BMT with GALT+ donor cells (1.12%)	1.23	82.71	169.87	23.98
483.11	GALT-null (no GFP)	BMT with GALT+ donor cells (0.01%)	1.76	67.49	130.52	31.42
483.10	GALT-null (no GFP)	BMT with GALT-null donor cells (88.6%)	9.72	44.78	91.87	17.76
