# Supplementary material for: A Pilot Study of Bone Marrow Transplantation in a GALT‐Null Rat Model of Classic Galactosemia
Source: JIMD Rep. 2025 Jul 11;66(4):e70037. doi: 10.1002/jmd2.70037 (PMC12254465; doi:10.1002/jmd2.70037)
Supplement: Supplementary file 7 — Table S4. Galactitol levels in RBC, plasma, liver, and brain samples from transplanted and control rats harvested at the 10‐week post‐transplant time point. [file JMD2-66-e70037-s006.pdf]

Supplemental Table 4: Galactitol levels in RBC, plasma, liver, and brain samples from transplanted and control rats harvested at the 10-week post-transplant time point.

Rat FKRC ID#	Recipient *GALT* and GFP genotypes	Treatment group (% GFP+ cells in blood at 10-weeks post-transplant)	Galactitol in RBC pmol/µL	Galactitol in plasma pmol/µL	Galactitol in liver pmol/µg	Galactitol in brain pmol/µg
478.01	Wild-type (no GFP)	no BMT (0.01%)	0.00	1.47	0.02	0.14
478.03	Wild-type (no GFP)	no BMT (5.3x10E-3%)	0.00	3.31	0.02	0.72
483.03	GALT-null (GFP+)	no BMT (94.7%)	4.47	3.67	54.21	21.82
483.05	GALT-null (GFP+)	no BMT (88.6%)	4.45	2.78	86.00	28.48
481.01	GALT-null (no GFP)	BMT with GALT+ donor cells (73.7%)	2.14	3.68	57.98	17.69
481.03	GALT-null (no GFP)	BMT with GALT+ donor cells (74.7%)	7.55	4.12	71.09	19.14
481.05	GALT-null (no GFP)	BMT with GALT+ donor cells (1.12%)	6.52	5.57	89.89	13.55
483.11	GALT-null (no GFP)	BMT with GALT+ donor cells (0.01%)	5.48	2.88	68.97	17.29
483.10	GALT-null (no GFP)	BMT with GALT-null donor cells (88.6%)	5.07	3.77	73.51	13.33
